# Porous Carbon Architecture Assembled by Cross-Linked Carbon Leaves with Implanted Atomic Cobalt for High-Performance Li–S Batteries

**DOI:** 10.1007/s40820-021-00676-6

**Published:** 2021-06-30

**Authors:** Ruirui Wang, Renbing Wu, Chaofan Ding, Ziliang Chen, Hongbin Xu, Yongfeng Liu, Jichao Zhang, Yuan Ha, Ben Fei, Hongge Pan

**Affiliations:** 1 Department of Materials Science, Fudan University, Shanghai, 200433 People’s Republic of China; 2 State Key Laboratory of Silicon Materials and School of Materials Science and Engineering, Zhejiang University, Hangzhou, 310027 People’s Republic of China; 3 Shanghai Synchrotron Radiation Facility, Zhangjiang Laboratory, Shanghai Advanced Research Institute, Chinese Academy of Sciences, Shanghai, 201204 People’s Republic of China; 4 Institute of Science and Technology for New Energy, Xi’an Technological University, Xi’an, 710021 People’s Republic of China

**Keywords:** Single-atom Co, 3D porous carbon architecture, Cathode, Lithium–sulfur battery

## Abstract

The practical application of lithium–sulfur batteries is severely hampered by the poor conductivity, polysulfide shuttle effect and sluggish reaction kinetics of sulfur cathodes. Herein, a hierarchically porous three-dimension (3D) carbon architecture assembled by cross-linked carbon leaves with implanted atomic Co–N_4_ has been delicately developed as an advanced sulfur host through a SiO_2_-mediated zeolitic imidazolate framework-L (ZIF-L) strategy. The unique 3D architectures not only provide a highly conductive network for fast electron transfer and buffer the volume change upon lithiation–delithiation process but also endow rich interface with full exposure of Co–N_4_ active sites to boost the lithium polysulfides adsorption and conversion. Owing to the accelerated kinetics and suppressed shuttle effect, the as-prepared sulfur cathode exhibits a superior electrochemical performance with a high reversible specific capacity of 695 mAh g^−1^ at 5 C and a low capacity fading rate of 0.053% per cycle over 500 cycles at 1 C. This work may provide a promising solution for the design of an advanced sulfur-based cathode toward high-performance Li–S batteries.

**Supplementary Information:**

The online version contains supplementary material available at 10.1007/s40820-021-00676-6.

## Highlights

- SiO_2_-mediated ZIF-L is developed to prepare Co–N_4_@2D/3D carbon.
- Co–N_4_@2D/3D integrates the advantages of 0D Co single atom and 2D/3D carbon support.
- Co–N_4_@2D/3D carbon-based sulfur cathode enables a high reversible specific capacity and low capacity fading rate.

**Supplementary Information:**

The online version contains supplementary material available at 10.1007/s40820-021-00676-6.

## Graphical abstract

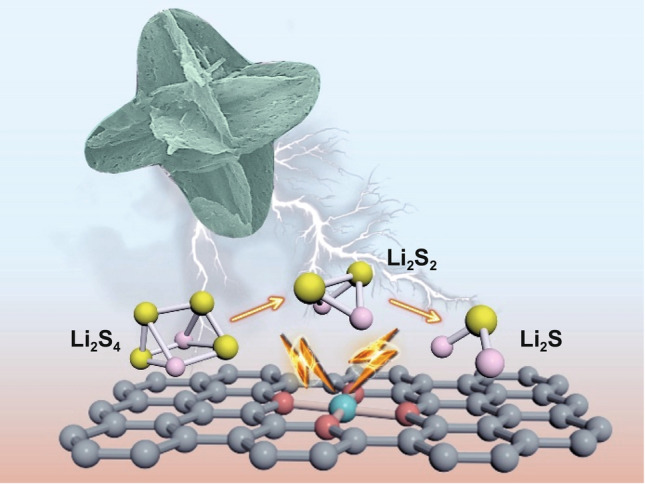

## Introduction

Nickel-metal hydride battery and lithium-ion battery have been widely used in portable electronic devices and energy storage systems [1–7]. Nevertheless, they are difficult to meet the ever-increasing demands on high energy density for future electric vehicles. Lithium–sulfur (Li–S) battery has been considered as one of the most promising next-generation energy storage systems due to the high energy density as well as resource abundance and environment benignity of the sulfur cathode [8–10]. However, the practical implementation of Li–S is seriously hindered by the sulfur cathode associated with poor electronic conductivity of sulfur, huge volume variation during operation, shuttle effect of intermediate lithium polysulfides (LiPSs) and sluggish LiPSs conversion kinetics, leading to inferior cycling stability and unsatisfactory coulombic efficiency [11–15].

Various strategies including designing advanced sulfur hosts, constructing functional separators and optimizing electrolyte additives have been proposed to address the above-mentioned problems [16–18]. Among them, encapsulating S particles into porous carbon matrix is deemed to be one of the most popular ways for enhancing the electrical conductivity of the cathode and alleviating the volume oscillation [19, 20]. Unfortunately, the carbon matrix is usually nonpolar, which shows unfavorable affinity to polar LiPSs and thus only functions as physical barrier to achieve a very limited LiPSs shutting inhibition. In this regard, polar species (e.g., non-metal heteroatom dopants, transition metal-based compounds and coordination polymers) with favorable affinity to LiPSs have been incorporated into carbon matrix to chemically interact with LiPSs and restrain its diffusion in the electrochemical process [21–24]. Nevertheless, these materials may suffer from various problems when used as sulfur host. Specifically, the non-metal heteroatom dopants have a limited effect for the LiPSs adsorption and conversion due to the small amount [25]. The transition metal-based compounds on carbon matrix are easily aggregated, while the coordination polymers have a weak conductivity and thus may block the electron/ion transfer and lower the kinetics of LiPSs conversion [26, 27].

Recently, atomically dispersed transition metal atoms anchored on nitrogen-doped carbon matrix (TM–N–C) have been demonstrated as a single-atom catalyst for Li–S batteries [28, 29]. Due to the maximum atom utilization and unique electronic structure, TM–N–C can not only mitigate LiPSs shutting through a strong chemical interaction but also efficiently facilitates the LiPSs conversion process via a catalytic effect [30, 31]. For example, Du et al. reported a synthesis of single-atom Co embedded in nitrogen-doped graphene (Co–N–C) and demonstrated that the Co–N–C was favorable for both the formation and decomposition of Li_2_S in lithiation and delithiation process [32]. Xie et al. implanted atomic Co dopants within mesoporous carbon and verified that they could serve as active sites to improve the interaction with LiPSs and accelerate the kinetics reactions of the sulfur redox [33]. Zhang et al. employed porous Mo–N–C nanosheets with atomically dispersed Mo–N_2_/C sites as a sulfur host to prepare cathode [34]. It was found that the Mo–N_2_/C could greatly decrease the energy barriers for the deposition and decomposition of Li_2_S, leading to improved LiPSs conversion kinetics and excellent electrochemical performance in Li–S battery. Despite these pioneer progress that has been made, the application of single-atom catalyst in Li–S batteries remain in its infancy. Moreover, most reported carbon matrix to support single-atom metal is usually limited to two-dimensional (2D) carbon nanosheets/graphene or three-dimension (3D) carbon spheres/polyhedra, and thus the exposure and utilization of active sites is still unsatisfactory.

Herein, for the first time, a 3D carbon architecture assembled by cross-linked 2D porous carbon leaves with atomically dispersed Co–N_4_ (Co–N_4_@2D/3D carbon) through a SiO_2_-mediated zeolitic imidazolate framework-L (ZIF-L) strategy is developed and employed as a multifunctional sulfur host toward Li–S batteries. Within this Co–N_4_@2D/3D carbon host, the 3D architecture can effectively prevent the stacking of 2D carbon subunits and ensure high structure stability, enabling a full exposure of active interfaces with richly accessible Co–N_4_ catalytic active sites and strong interaction with LiPSs. Meanwhile, the porous 2D carbon leaves not only allow abundant space for the volume variation of sulfur but also provide a rapid electron/ion transfer pathway in collaboration with a cross-linked assembly way, further contributing to facile sulfur redox kinetics and stable sulfur electrochemistry. As a result, the sulfur-loaded Co–N_4_@2D/3D carbon cathodes could achieve a superior rate capability (695 mAh g^−1^ at 5 C) and long-term cycling stability (0.053% decay rate per cycle after 500 cycles at 1 C).

## Experiment Section

### Synthesis of Co/Zn-ZIF-L

First, 40 mg of Co(acac)_2_·6H_2_O, 820 mg of Zn(NO_3_)_2_·6H_2_O and 1.64 g of 2-methylimidazole were dissolved into 80 mL of DI water, followed by stirring for 30 min. Afterward, the solution was placed at room temperature for 4 h. The resultant Co/Zn-ZIF-L precursor was obtained by centrifugation, washed with DI water and dried in a vacuum at 60 °C.

### Synthesis of Co/Zn-ZIF-L@SiO_2_

In a typical synthesis, 600 mg of Co/Zn-ZIF-L powder was added into 240 mL of DI water in the presence of cetyltrimethylammonium bromide (CTAB) (150 mg) and NaOH (57.6 mg). Afterward, 6 mL of methanol dissolved with 1.5 mL of tetraethyl orthosilicate was drop-wise added into the above solution and then stirred for 30 min. The Co/Zn-ZIF-L@SiO_2_ was obtained after collecting the precipitate and dried at 60 °C for 12 h.

### Preparation of Co–N_4_@2D/3D Carbon

A clean porcelain boat loaded with Co/Zn-ZIF-L-@SiO_2_ was heated to 930 °C with a ramp rate of 2 °C min^−1^ and maintained at this temperature for 3 h under the flowing nitrogen to obtain Co–N_4_@2D/3D carbon@SiO_2_. Finally, the SiO_2_ coating was etched by 1 M HF to generate Co–N_4_@2D/3D carbon composites. The preparation of nitrogen-doped carbon with implanted cobalt single atoms (Co–N_4_@carbon) was similar to that for making Co–N_4_@2D/3D carbon except no SiO_2_ coating. The preparation of 2D nitrogen-doped carbon leaves (2D carbon) was similar to that for making Co–N_4_@2D/3D carbon except no adding Co(acac)_2_·6H_2_O.

### Preparation of S@Co–N_4_@2D/3D Carbon Composites

The preparation of S@Co–N_4_@2D/3D carbon composites was achieved by a melt-diffusion method. In a typical process, the sulfur powder and the Co–N_4_@2D/3D carbon were ground together in a mass ratio of 3:1 and treated at 155 °C for 12 h in a sealed autoclave. After that, the obtained mixture was placed in a tube furnace and heated to 230 °C for 30 min under the flowing nitrogen to obtain the resulting S@Co–N_4_@2D/3D carbon composites.

### Materials Characterizations

The crystal structures of products were characterized by X-ray diffractometer (XRD) in D8 ADVANCE with Cu Kα radiation. Field-emission scanning electron microscope (FESEM, ZEIS, Ultra-55) and transmission electron microscope (TEM, JEOL-2100) characterizations were performed to investigate the morphology and microstructure of samples. X-ray photoelectron spectroscopy (XPS) measurement was conducted to identify surface states of products on Thermo ESCALAB 250XI (Mg Kα radiation source). The element contents of products were determined through Thermogravimetric analysis (METTLER TOLEDO TGA2). The porosity of samples was investigated through N_2_ sorption measurements on Autosorb IQ Gas Sorption instrument.

### Electrochemical Measurements

The 2032-type coin half-cells were assembled in a glove-box (H_2_O and O_2_ < 0.1 ppm) filled with Ar. S@Co–N_4_@2D/3D carbon, Celgard 2400 membrane and Li metal foil were used as active material, separator and counter electrode, respectively. 1 M lithium bis-trifluoromethanesulfonimide (LiTFSI) in a mixture of DOL and DME (1:1 in volume) with 2.0 wt% LiNO_3_ additive was used as the electrolyte. The Galvanostatic charge–discharge test was carried out over the voltage range of 1.7–2.8 V on a Land-CT2001A battery test system at room temperature. The cyclic voltammetry (CV) curves were collected over the same potential at 0.1 mV s^–1^ on AutoLab-PGSTAT302N electrochemical work station. The electrochemical impedance spectroscopy (EIS) was also examined in a 0.1 MHz–0.01 Hz frequency range on above workstation.

### Adsorption Test of Lithium Polysulfide

0.2 M Li_2_S_6_ solution was prepared by dissolving a certain amount of S and Li_2_S (1:5 by molar ratio) in 1,2-dioxolane (DOL)/dimethoxymethane (DME) (1:1 in volume). The adsorption tests were carried out by adding 10 mg of Co–N_4_@2D/3D carbon, Co–N_4_@carbon and 2D carbon into 3 mL of diluting Li_2_S_6_ solution, respectively. After standing for 12 h, the color change of solution was observed. In addition, the supernatant solution was used for ultraviolet–visible (UV–Vis) measurement.

### Theoretical Calculation

The calculations based-on density functional theory (DFT) were carried out using projector augmented wave (PAW) method in Vienna ab initio simulation package (VASP). The *k*-points were sampled using the Monkhorst–Pack mesh for all slab models. All models were optimized from a supercell of graphene containing 4 × 4-unit cells. The cut off energy for plane-wave basis set was 520 eV, and a vacuum layer of about 20 Å in thickness was introduced for all the surfaces. The total energy convergence was set to be lower than 1 × 10^−5^ eV with the force convergence set at 0.01 eV Å^−1^ for geometric optimization.

## Results and Discussion

### Morphological and Microstructural Characterizations

**Fig. 1:**
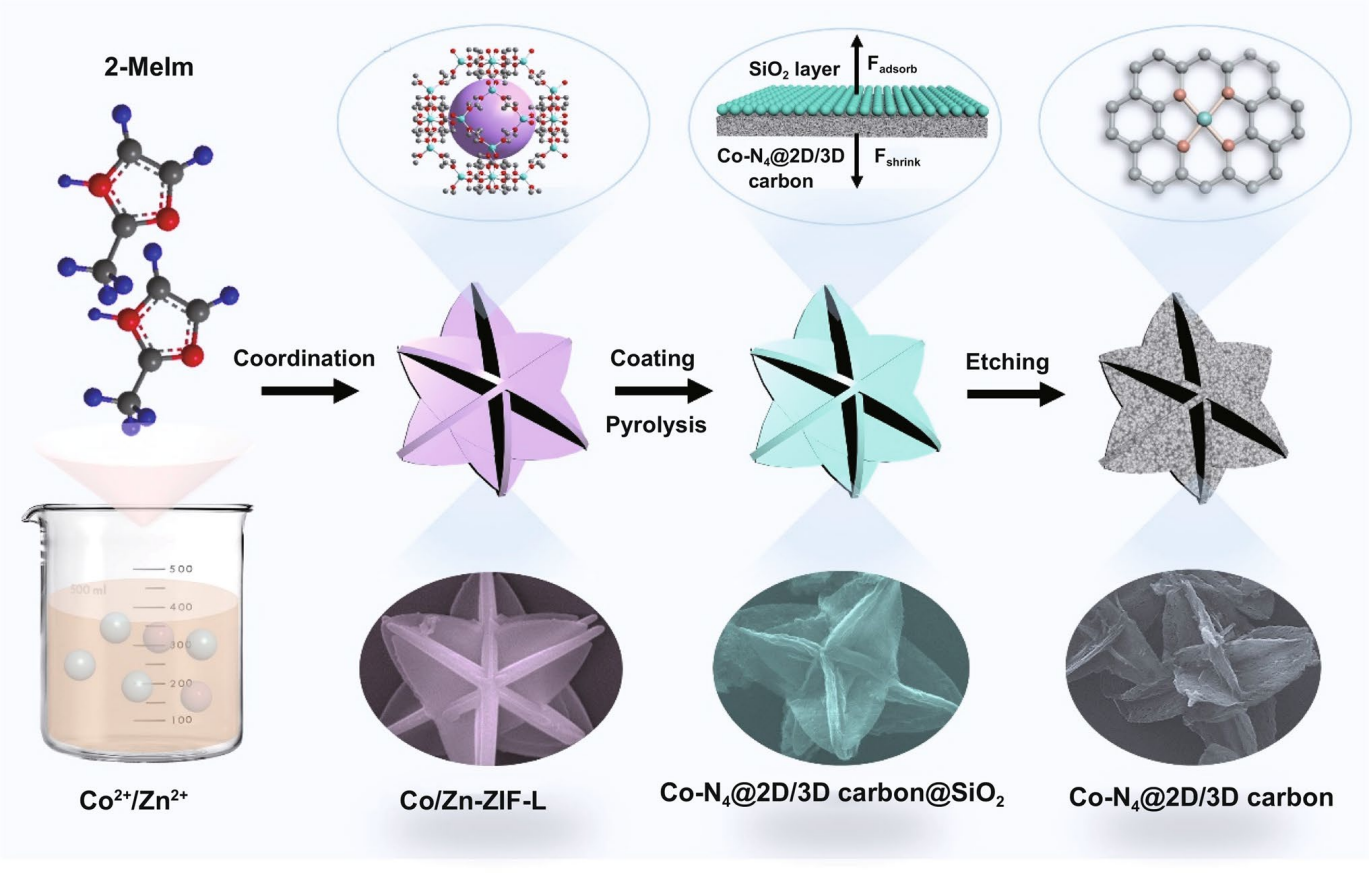
Schematic synthesis procedure of Co–N_4_@2D/3D carbon

Figure 1 schematically illustrates the synthetic route of Co–N_4_@2D/3D carbon composites. First, 3D architectures assembled by cross-linked 2D ZnCo–based zeolitic imidazolate framework (ZIF)-L leaves were synthesized by the coordination reaction of Co^2+^/Zn^2+^ ions and 2-methylimidazole ligands in water and employed as template materials. The X-ray powder diffraction patterns of the as-synthesized Co/Zn-ZIF-L matched well with that of the simulated Zn-ZIF-L, indicating that the introduced Co^2+^ dopants may partially substitute the original Zn^2+^ ions in Zn-ZIF-L (Fig. S1). Field-emission scanning electron microscopy (FESEM) observation further showed that Co/Zn-ZIF-L architecture was constructed by cross-linked leaf-like nanosheets with a a relatively uniform dimensions and morphology (Figs. S2 and S3a, b). Subsequently, amorphous SiO_2_ was evenly coated on the surface of architecture template by a hydrolysis of tetraethyl orthosilicate process to form Co/Zn-ZIF-L@SiO_2_ core–shell particles (Fig. S4). After a pyrolysis process under flowing N_2_ at 930 °C, the Co–N_4_@2D/3D carbon@SiO_2_ was obtained. As characterized by FESEM image in Fig. S3c and 3*d* (Supporting Information), the Co–N_4_@2D/3D carbon@SiO_2_ sample could retain the morphology of the template except that its surface became rough and has slight shrinkage. During this process, the organic ligands liberated from Co/Zn-ZIF-L were carbonized into N-doped porous carbon, while the low boiling-point Zn atoms were evaporated, and the high boiling-point Co atoms remained and were anchored on the carbon matrix. After removing the SiO_2_ shell using HF, the final Co–N_4_@2D/3D carbon was obtained. It can be observed that Co–N_4_@2D/3D carbon remained the initial 3D structure but bestrewed with numbers of grooves and mesopores on the surface of nanosheets (Fig. S3e, f). Note that the presence of SiO_2_ coating not only created more pores within the carbon architecture but also could allow an anisotropic thermal shrinkage of Co/Zn-ZIF-L to preserve the configuration of template at high temperature.

For comparison, Co/Zn-ZIF-L without SiO_2_ coating and Zn-ZIF-L@SiO_2_ were employed as precursor to fabricate Co–N_4_@carbon and 2D carbon leaves, respectively. As shown in Fig. S5a, b, Co–N_4_@carbon was aggregated, and no obvious pores could be observed on its surface. Figure S6a, b shows the FESEM images of 2D carbon leaves, from which porous nanosheets stacked together.

**Fig. 2:**
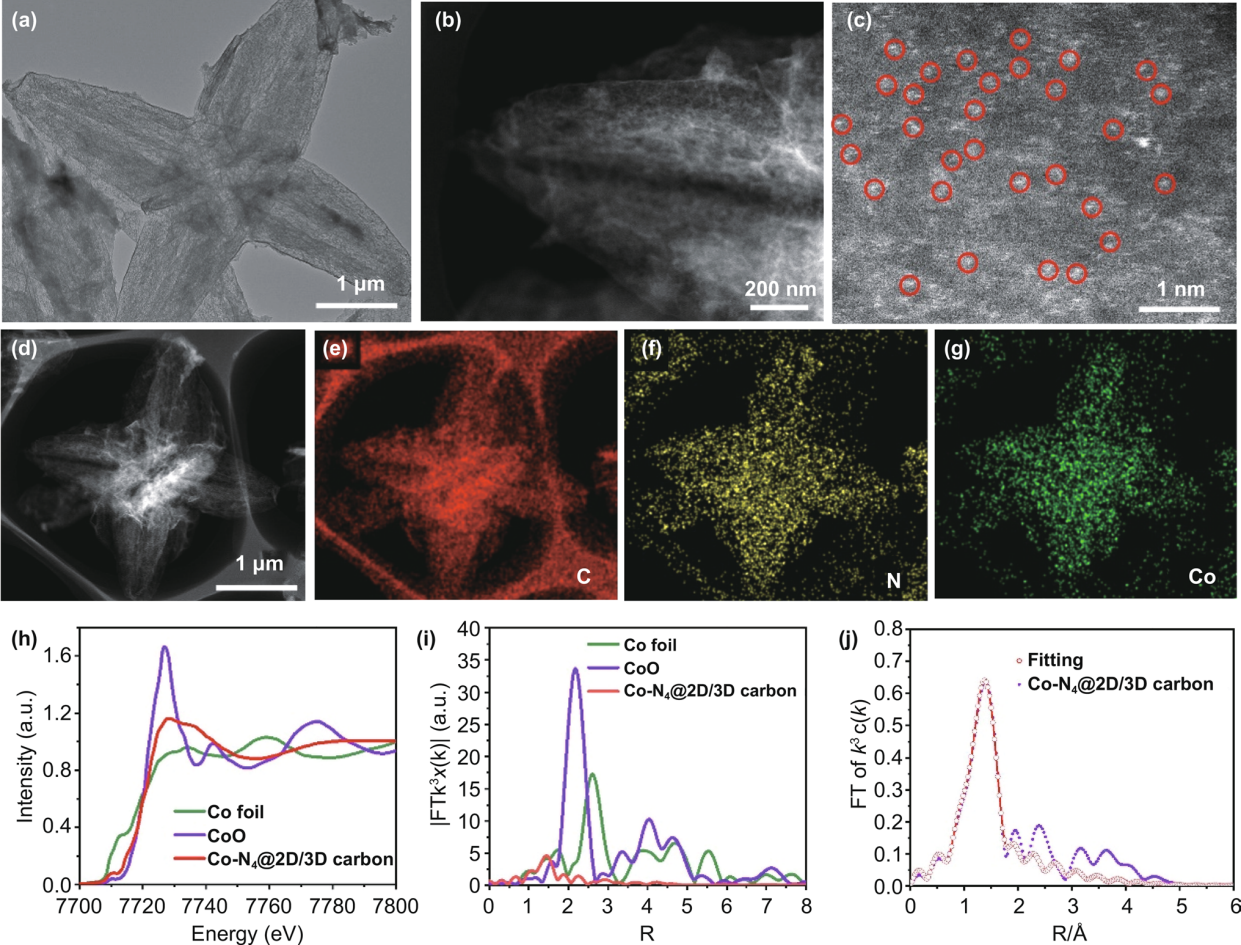
**a** TEM image, **b–d** HAADF-STEM images of Co–N_4_@2D/3D carbon and **e–g** corresponding element mapping of C, N and Co in Co–N_4_@2D/3D carbon. **h** Co K-edge XANES spectra and **i** Fourier transform (FT) of the Co–K-edge for Co–N_4_@2D/3D carbon, CoO and Co foil. **j** The corresponding EXAFS r space fitting curve of Co–N_4_@2D/3D carbon

The microstructure of the obtained samples was further investigated by transmission electron microscope (TEM). As shown in Fig. 2a, the Co–N_4_@2D/3D carbon displayed a 3D architecture assembled by cross-linked 2D porous carbon leaves, while Co–N_4_@carbon showed an irregular morphology without obvious pores (Fig. S5c, d). The implanted Co single atoms in Co–N_4_@2D/3D carbon were verified by atomic-resolution high angle annular dark-field scanning transmission electron microscope (HAADF-STEM). As shown in Fig. 2b, c, dense isolated bright dots could be observed, meaning that high-density single Co atoms homogeneously distributed in 2D/3D porous carbon. The energy-dispersive spectroscopy (EDS) results shown in Fig. 2d–g revealed the uniform distribution of C, N and Co elements within the 3D architecture framework. The Co contents in the Co–N_4_@2D/3D carbon and Co–N_4_@carbon were about 1.01% and 1.33%, respectively, according to the inductively coupled plasma-optical emission spectroscopy (ICP-OES) results.

### Phase, Coordination Environment and Surface Property Characterizations

Figure S7 shows the XRD patterns of Co–N_4_@2D/3D carbon and Co–N_4_@carbon, in which no characteristics peaks for Co species were found, revealing that Co is atomically dispersed rather than aggregated nanoparticles on them. The chemical states and local environment of Co sites for Co–N_4_@2D/3D carbon were analyzed by synchrotron-based X-ray absorption near-edge structure (XANES) spectroscopy and extended X-ray absorption fine structure (EXAFS) spectroscopy. The XANES spectra of Co K-edge show a near-edge absorption energy of Co–N_4_@2D/3D carbon located between those of the Co foil and CoO (Fig. 2h), implying that Co single atoms carried positive charges [35, 36]. The *k*_2_-weighted Fourier transforms (FT) of the extended EXAFS in Fig. 2i demonstrated a main peak at 1.4 Å for Co–N_4_@2D/3D carbon, which was attributed to Co–N scattering path [37, 38]. The absence of longer Co–Co backscattering paths in the spectra of Co–N_4_@2D/3D carbon further suggests the atomic dispersion of Co atoms, in line with the STEM founding. The quantitative structural parameters of Co in the Co–N_4_@2D/3D carbon were determined by least-squares EXAFS fitting shown in Fig. 2j and Table S1. The best fitting result for the first shell indicates that each Co atom is likely coordinated by four nitrogen atoms, suggesting a Co–N_4_ configuration for the Co–N bonding [39, 40].

**Fig. 3:**
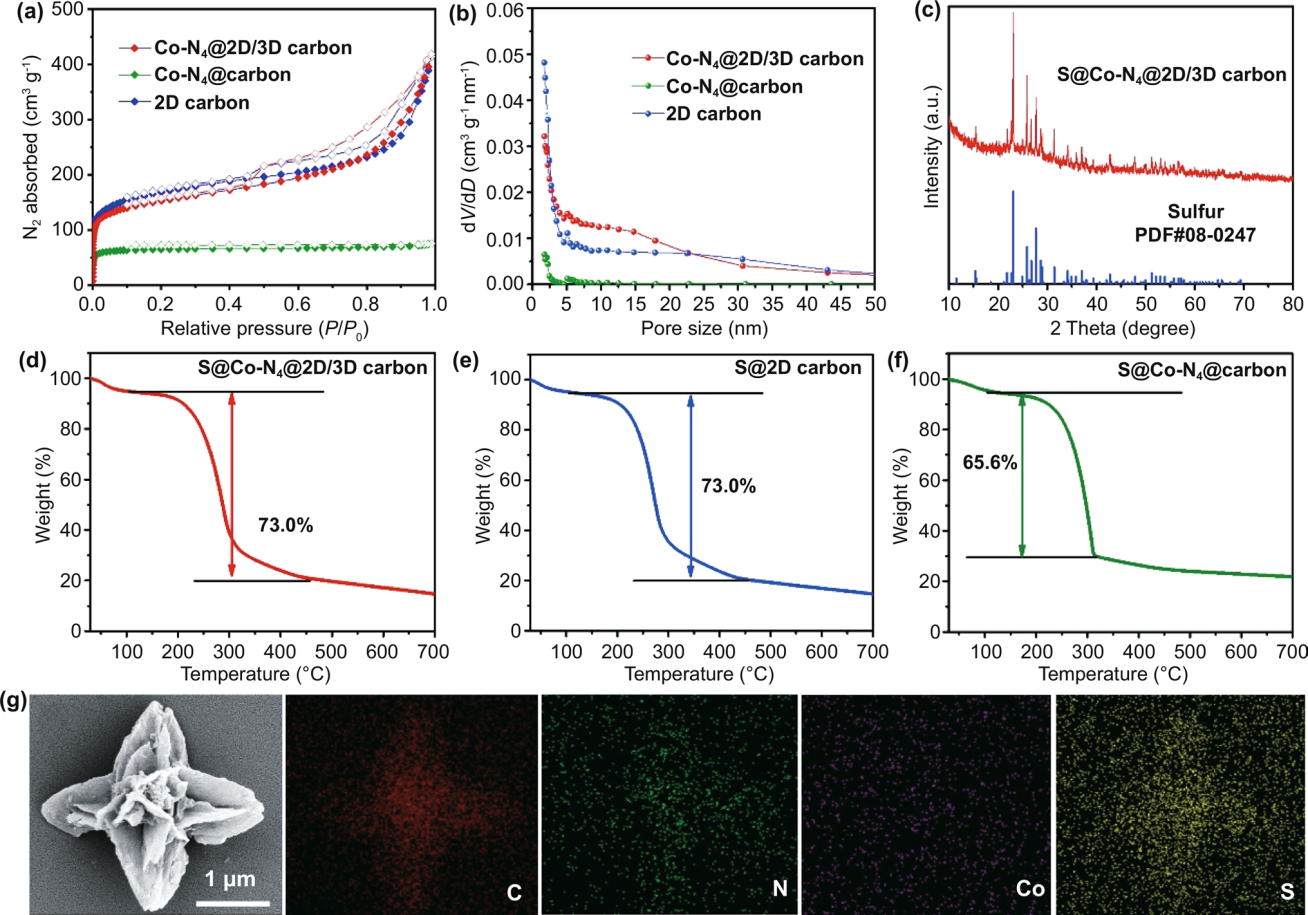
**a** N_2_ sorption isotherms based on the Barrett–Joyner–Halenda (BJH) method and **b** the pores distribution of Co–N_4_@2D/3D carbon, Co–N_4_@carbon and 2D carbon. **c** XRD pattern of S@Co–N_4_@2D/3D carbon. TGA curves of **d** S@Co–N_4_@2D/3D carbon, **e** S@2D carbon and **f** S@Co–N_4_@carbon. **g** FESEM image of S@Co–N_4_@2D/3D carbon composites and corresponding elements mapping images of C, N, Co and S

The pore structure of samples was investigated by nitrogen sorption isotherms. As shown in Fig. 3a, b, Co–N_4_@2D/3D carbon exhibited a high Brunauer–Emmett–Teller (BET) surface area of 482 m^2^ g^−1^ and large pore volume of 0.649 cm^3^ g^−1^, which were similar with those of 2D carbon (534 m^2^ g^−1^ and 0.641 cm^3^ g^−1^) but significantly larger than those of Co–N_4_@carbon (198 m^2^ g^−1^ and 0.117 cm^3^ g^−1^). The above results illustrated that the introduction of SiO_2_ would prevent the stacking of 2D carbon leaves during thermal treatment and was favorable to higher pore volume, which are consistent with FESEM observations. Additionally, the implanted atomically dispersed Co–N_4_ in Co–N_4_@2D/3D carbon may not block the formation of mesoporous structure and have a negligible effect on the surface area of composites. The high specific surface area of Co–N_4_@2D/3D carbon composites provide rich electrochemical active interfaces to accelerate the sulfur redox reaction, while the large pore volume is benefiting to accommodating the sulfur species and buffering the volume variation. Encouraged by the chemical and microstructure traits, the Co–N_4_@2D/3D carbon composite was employed as sulfur host to fabricate S@Co–N_4_@2D/3D carbon composites through a melt-diffusion method. After sulfur infiltration, the diffraction peaks of sulfur can be identified in the XRD patterns of S@Co–N_4_@2D/3D carbon composites (Fig. 3c). The sulfur content of S@Co–N_4_@2D/3D carbon composites is measured to be 73 wt% by the thermogravimetric analysis (TGA), while the sulfur content of S@Co–N_4_@carbon composites through the same melt-diffusion process is only 65.6%, further signifying the advantages of Co–N_4_@2D/3D carbon host (Fig. 3d). FESEM image in Fig. 3e showed that the obtained S@Co–N_4_@2D/3D carbon composite preserves the initial 3D architecture morphology and there is no any aggregation of sulfur particles on its surface. Meanwhile, the corresponding elements mapping revealed a homogeneous distribution of C, N, Co and S element within the architecture, illustrating the full incorporation of sulfur into the Co–N_4_@2D/3D carbon host (Fig. 3f).

### Adsorption Ability and Catalytic Effect

**Fig. 4:**
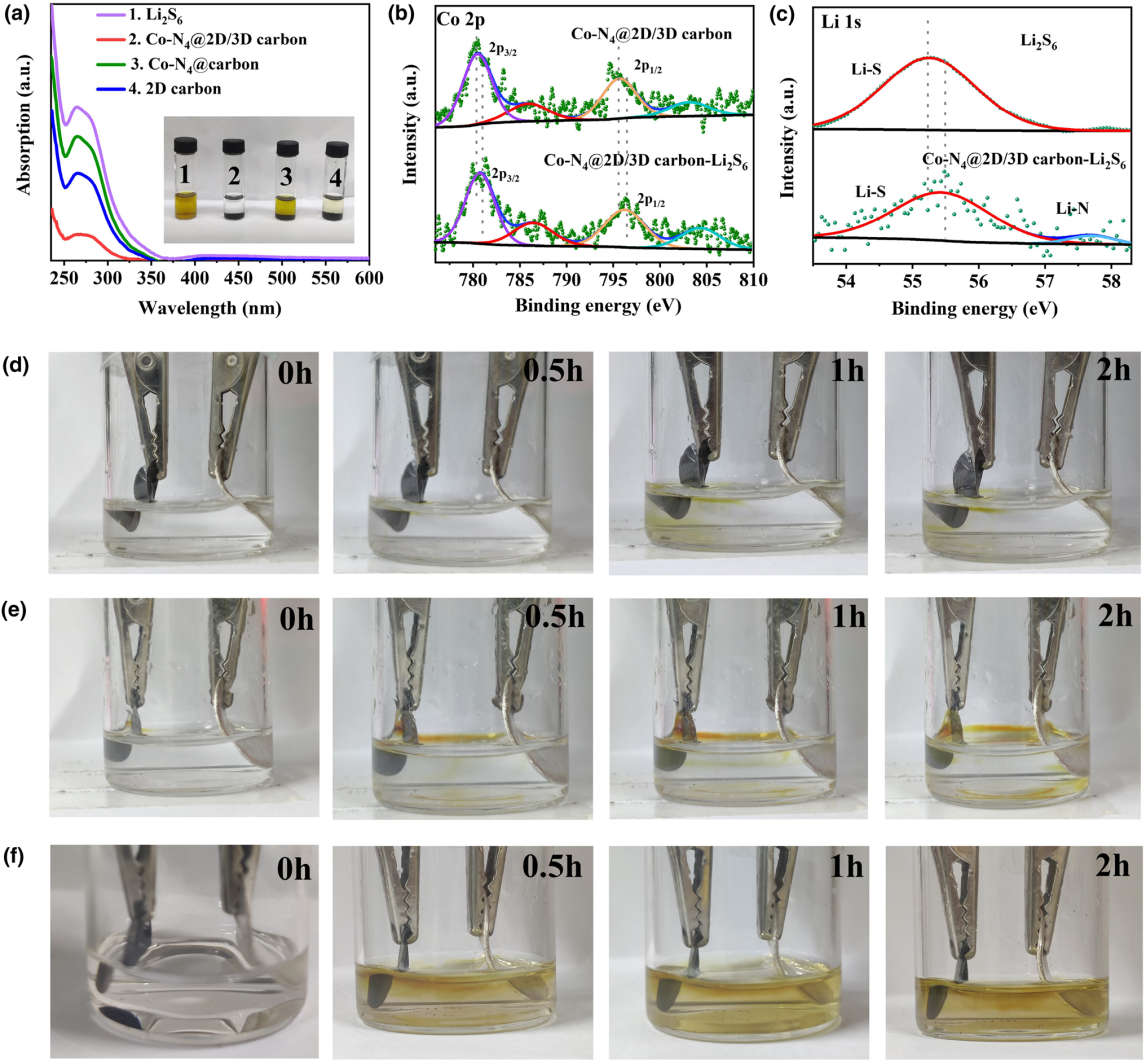
**a** UV–Vis absorption spectra and optical images of pure Li_2_S_6_ solutions as well as the Li_2_S_6_ solutions trapped by Co–N_4_@2D/3D carbon, Co–N_4_@carbon and 2D carbon. **b** The Co 2*p* XPS of spectra of Co–N_4_@2D/3D before and after adsorbing Li_2_S_6_. **c** the Li 1*s* XPS spectra of Li_2_S_6_ before and after been adsorbed with Co–N_4_@2D/3D carbon. In-situ beaker cell observation during discharging from 2.8 to 1.7 V at 0.1 C for **d** S@Co–N_4_@2D/3D carbon, **e** S@2D carbon, and **f** S@Co–N_4_@carbon

To investigate the adsorption properties of Co–N_4_@2D/3D carbon with the LiPS, visualized absorption experiments were performed by adding Co–N_4_@2D/3D carbon, Co–N_4_@carbon, and 2D carbon into an as-prepared Li_2_S_6_ solution. After 12 h, the Li_2_S_6_ solution containing Co–N_4_@2D/3D carbon became almost colorless, while the light-yellow color only slightly faded in the reference Li_2_S_6_ solution containing Co–N_4_@carbon and 2D carbon (Fig. 4a inset), indicating a strong trapping capability of Co–N_4_@2D/3D carbon for long-chain LiPSs. The concentration changes of LiPSs after adding samples were further analyzed by ultraviolet–visible (UV) absorption. The S_6_^2−^ species for Co–N_4_@2D/3D carbon exhibited a much lower peak intensity compared with other samples, which is consistent with the result of LiPS adsorption test (Fig. 4a). To further study the chemical interaction between Co–N_4_@2D/3D carbon and Li_2_S_6_, XPS characterizations were conducted on the Co–N_4_@2D/3D carbon and Li_2_S_6_ before and after adsorption. As shown in Fig. 4b, after adsorbing Li_2_S_6_, the peaks of Co 2*p* 3/2 located at 780.45 and 785.92 eV shift to higher binding energy centered at 780.74 and 786.46 eV, respectively, while two Co 2*p* 1/2 peaks at 795.64 and 803.12 shift to 796.33 eV and 804.44 eV, respectively, revealing that the electron transfer from Li_2_S_6_ to Co [11, 41]. Moreover, Li 1*s* peak of Co–N_4_@2D/3D carbon-Li_2_S_6_ shifts 0.39 eV toward higher binding energy, accompanied with an additional peak (57.9 eV) (Fig. 4c), indicating the formation of Li–N bond [42, 43]. These results demonstrate that Co–N_4_@2D/3D carbon can effectively escape soluble LiPSs into the electrolyte and ameliorate weight loss of active species.

An in-situ beaker cell observation experiment was also conducted to study the polysulfides diffusion in electrolyte for S@Co–N_4_@2D/3D carbon, S@2D carbon and S@Co–N_4_@carbon during electrochemical reaction. The assembled cell was first discharged at 0.1 C. As shown in Fig. 4e, f, the color of electrolyte in the S@2D carbon- and S@Co–N_4_@carbon-based cell changed from colorless to yellow with the reaction going on, implying the severe dissolution of LiPSs intermediates into electrolyte before transforming into insoluble Li_2_S. In contrast, the color of electrolyte in the S@Co–N_4_@2D/3D carbon-based cell underwent a negligible change (Fig. 4d), illustrating the strong absorptivity of Co–N_4_@2D/3D carbon for dissoluble LiPSs.

**Fig. 5:**
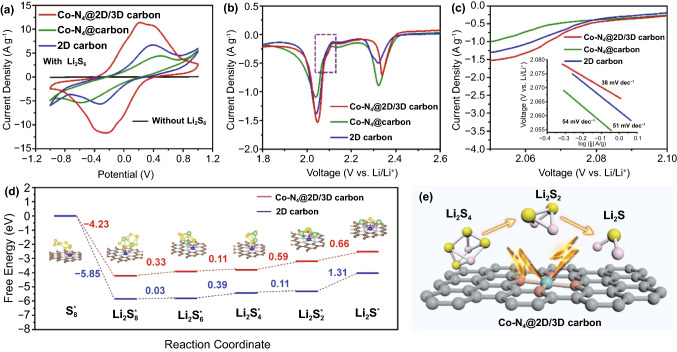
**a** CV curves of symmetric cells from − 1 to 1 V and **b** LSV curves between 1.7 and 2.8 V at a scan rate of 0.1 mV s^−1^ with the Co–N_4_@2D/3D carbon, Co–N_4_@carbon and 2D carbon electrodes. **c** Potentiostatic polarization curves from the LSV measurements and the inset showing the derived Tafel plots. **d** Energy profiles for the reduction of LiPSs on Co–N_4_@2D/3D carbon and 2D carbon substrates, the inset showing the optimized adsorption conformations of intermediate species on Co–N_4_@2D/3D carbon and 2D carbon substrates. **f** Schematic illustration of the catalysis effect of Co–N_4_@2D/3D carbon toward LiPSs

To reveal the catalytic effect of Co–N_4_@2D/3D carbon on enhancing the kinetics of LiPSs conversion in the Li–S battery, cyclic voltammetry (CV) measurements were carried out in the voltage from − 1 to 1 V for the symmetric cells with using Co–N_4_@2D/3D carbon as both work and counter electrodes and 0.2 M Li_2_S_6_ as the electrolyte. As shown in Fig. 5a, Co–N_4_@2D/3D carbon electrode exhibited a stronger peak current density and voltage hysteresis than the Co–N_4_@carbon and 2D carbon electrodes, indicating the higher electrochemical reversibility and better catalytic reaction kinetics of the Co–N_4_@2D/3D carbon. The excellent catalytic activity could be further confirmed by linear sweep voltammetry (LSV) tests. As shown in Fig. 5b, the intensity of peak corresponding to the conversion reaction from Li_2_S_4_ to Li_2_S for Co–N_4_@2D/3D carbon was larger those of Co–N_4_@carbon and 2D carbon, indicating its superior reaction kinetics. Meanwhile, the Tafel plots derived from this reaction (marked by the purple liner in Fig. 5b) reveal the smallest Tafel slope (38 mV dec^−1^) for Co–N_4_@2D/3D carbon compared with those for Co–N_4_@carbon (54 mV dec^−1^) and 2D carbon (51 mV dec^−1^) (Fig. 5c), indicated that the enhancement in sulfur reaction kinetics can be ascribed to the hierarchical architecture, which provides more accessible active sites to accelerate the conversion from Li_2_S_4_ to Li_2_S and thus improves the utilization of sulfur.

A potentiostatic nucleation measurement was conducted to further demonstrate the advantage of Co–N_4_@2D/3D for the conversion from liquid LiPSs to solid Li_2_S through using identical carbon fibers loaded with Co–N_4_@2D/3D carbon, Co–N_4_@carbon and 2D carbon electrodes. As shown in Fig. S8 (Supporting Information), the capacities of Li_2_S precipitation on Co–N_4_@2D/3D carbon, Co–N_4_@carbon and 2D carbon were calculated to be 217.01, 163.19, and 182.07 mAh g^−1^, respectively, manifesting the highest catalytic activity of Co–N_4_@2D/3D carbon toward Li_2_S precipitation. The CV curves of S@Co–N_4_@2D/3D carbon, S@Co–N_4_@carbon and S@2D carbon cathodes at different scan rates (0.1–0.6 mV s^−1^) were shown in Fig. S9a, c and e. The well-separated cathodic/anodic peaks with a larger current density reveal the mitigated electrochemical polarization of the S@Co–N_4_@2D/3D carbon in comparison with other electrodes. Accordingly, a linear relationship between the anodic and cathodic peak currents and square root was also investigated (Fig. S9b, d and f), suggesting the diffusion-limited process of cathodes. Moreover, according to the fitting of this linear dependence, Li^+^ diffusion coefficient (*D*) can be calculated using the Randles–Sevcik equation [44, 45]:

1 $$I_{{\text{p}}} = (2.69 \times 10^{5} ) \times n^{{1.5}} AD^{{0.5}} Cv^{{0.5}}$$

where *I*_p_ represents the peak current density (A), *n* is the number of transferred charges, *A* is the area of the electrode (cm^2^), *C* is the concentration of lithium ions in the electrolyte (mol cm^−3^), and *ν* is the scan rate (V s^−1^). The values of *D* for S@Co–N_4_@2D/3D carbon at peaks 1, 2, and 3 are calculated to be 3.20 × 10^−8^, 3.88 × 10^−8^, and 1.06 × 10^−7^ cm^2^ s^−1^, respectively, which are higher than those of S@Co–N_4_@carbon and S@2D carbon cathodes, suggesting the facilitated ion transfer of S@Co–N_4_@2D/3D carbon.

The improved reaction kinetics and chemical interaction between the S@Co–N_4_@2D/3D carbon cathode and LiPS could be further understood by first-principles calculations. The optimized configurations of Co–N_4_@2D/3D carbon and 2D carbon were shown in Fig. S10. As shown in Fig. S11, the Co–N_4_@2D/3D carbon has much higher adsorption energy for Li_2_S_4_ and Li_2_S_6_ (− 0.927 and − 0.904 eV) than 2D carbon (− 0.366 and − 0.274 eV). Figure 5d displays the calculated Gibbs free energy of the different possible reactions from S_8_ to Li_2_S on the Co–N_4_@2D/3D carbon and 2D/3D carbon during the electrochemical process [46, 47]. It can be found that the conversion from S_8_ to Li_2_S_8_ is exothermic and occurs spontaneous, while the subsequent four reduction steps for the formation of Li_2_S_6_, Li_2_S_4_, Li_2_S_2_, and Li_2_S are all endothermic. Due to the largest positive Gibbs energy barrier, the conversion from Li_2_S_2_ to Li_2_S is the rate-determining step in the whole process [32, 48]. For this rate-determining step, the Gibbs free energy of Co–N_4_@2D/3D carbon support is 0.66 eV, much lower than that of 2D carbon support (1.31 eV), suggesting that the reduction in sulfur is thermodynamically more favorable on Co–N_4_@2D/3D carbon than on 2D carbon. Figure 5e schematically illustrated the catalysis effect of Co–N_4_@2D/3D carbon toward lithium polysulfides, from which fully accessible Co–N_4_ sites implanted within 2D/3D carbon framework can significantly facilitate the redox reaction from Li_2_S_4_ to Li_2_S and thus dramatically enhance the electrochemical performance of sulfur cathodes.

### Electrochemical Performance Evaluation

**Fig. 6:**
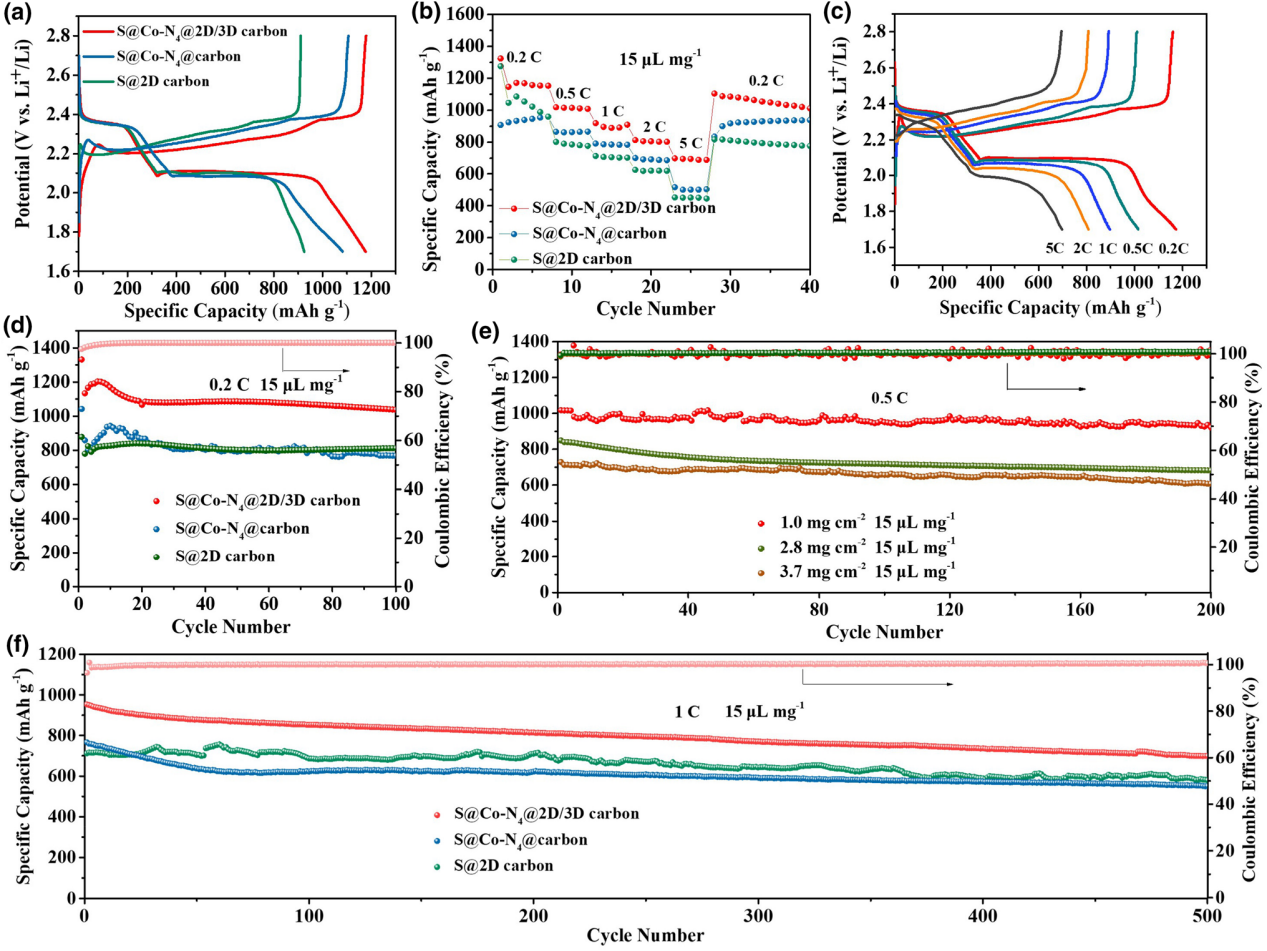
**a** Charge–discharge profiles at 0.2 C and **b** rate performance at various current rate of S@Co–N_4_@2D/3D carbon, S@Co–N_4_@carbon and S@2D carbon electrodes. **c** The charge–discharge profiles of the S@Co–N_4_@2D/3D carbon electrode at different current rates. **d** Cycling stability at 0.2 C and **f** cycling stability at 1 C for S@Co–N_4_@2D/3D carbon, S@Co–N_4_@carbon and S@2D carbon electrodes. **e** Cycling performance of S@Co–N_4_@2D/3D carbon with sulfur loading of 1.0, 2.8, and 3.7 mg cm^−2^ at 0.5 C

To systematically evaluate the electrochemical performance of the S@Co–N_4_@2D/3D carbon cathode, coin-type Li–S batteries were assembled. The galvanostatic charge–discharge profiles of S@Co–N_4_@2D/3D carbon as well as contrastive samples were first investigated. As shown in Fig. 6a, all cathodes display two discharge plateaus about at 2.3 and 2.1 V in discharge process, which could be ascribed to the electroreduction of S_8_ into long-chain LiPSs (Li_2_S_8_ and Li_2_S_6_) and further reduction in the LiPSs to Li_2_S_2_ and Li_2_S [49, 50]. Nevertheless, the S@Co–N_4_@2D/3D carbon cathode displayed the highest discharge capacity and the smallest potential polarization. Meanwhile, the CVs of S@Co–N_4_@2D/3D carbon, S@Co–N_4_@carbon and S@2D carbon cathodes were collected within a potential window of 1.7–2.8 V at a scan rate of 0.1 mV s^−1^ (Fig. S12). It is observed that the S@Co–N_4_@2D/3D carbon displays higher peak intensity and lower voltage gap than contrast electrodes. These results suggest the fastest conversion kinetics for LiPS because of the high affinity and catalytic activity of Co–N_4_@2D/3D carbon.

The rate performance of S@Co–N_4_@2D/3D carbon, S@Co–N_4_@carbon and S@2D carbon electrodes were assessed under various current rates from 0.2 to 5 C with an electrolytes/sulfur (E/S) ratio of 15 µL mg^−1^. As shown in Fig. 6b, when the current densities are 0.2 C, 0.5 C, 1 C, 2 C, and 5 C, the average specific capacities of the S@Co–N_4_@2D/3D carbon cathode are 1171, 1015, 909, 805, and 695 mAh g^−1^, respectively, much better than those of S@Co–N_4_@carbon and S@2D carbon electrodes. When the current density is restored to 0.2 C, a high specific capacity of 1064 mAh g^−1^ is recovered, manifesting a superior rate capability of S@Co–N_4_@2D/3D carbon cathode [49]. Figure 6c shows the charge/discharge profiles of S@Co–N_4_@2D/3D carbon cathode at different current densities. With the increase in the current density, the potential difference between the charge and discharge plateaus increases gradually. However, the two well-maintained plateaus can still be observed even at 5 C, implying the fast reaction kinetics in S@Co–N_4_@2D/3D carbon cathode. Electrochemical impedance spectroscopy (EIS) measurements of the symmetric cells were carried out to further evaluate the kinetics reactions at the electrode interface. As shown in Fig. S13, the charge transfer resistance at the S@Co–N_4_@2D/3D carbon cathode/polysulfide interface is smaller than those of the S@Co–N_4_@carbon and S@2D carbon [51–54], revealing the favorable enhancement of the charge transfer during the polysulfide conversion reaction because of the synergistic effect of 2D/3D architecture and atomically dispersed Co–N_4_.

Figure 6d shows the cycling stability of the S@Co–N_4_@2D/3D carbon, S@Co–N_4_@carbon and S@2D carbon cathode with an E/S ratio of 15 µL mg^−1^. After 100 cycles at a current density of 0.2 C, the S@Co–N_4_@2D/3D carbon electrode could still maintain a high discharge capacity of 1000 mA h g^−1^ with 83.3% capacity retention and a Coulombic efficiency of nearly 100%, much higher than those of S@Co–N_4_@carbon and S@2D carbon. Figure 6e further displays the cycling stability of S@Co–N_4_@2D/3D carbon electrode with different sulfur loading density at a larger density of 0.5 C with an E/S ratio of 15 µL mg^−1^. The S@Co–N_4_@2D/3D carbon electrode can deliver initial capacities of 850 and 728 mAh g^−1^ and still maintain reversible capacities of 682 and 607 mAh g^−1^ even after 200 cycles at high sulfur loadings of 2.8 and 3.7 m cm^−2^, respectively. Meanwhile, the S@Co–N_4_@2D/3D carbon cathode with a higher sulfur loading (4.6 mg cm^−2^) and a lower E/S ratio of 8 µL mg^−1^ was also investigated. As shown in Fig. S14, it could maintain the areal capacity as high as 3.6 mAh cm^−2^ after 100 cycles at 0.2 C, further demonstrating the feasibility of Co–N_4_@2D/3D carbon as a sulfur host for practical Li–S batteries. To further illustrate the advantage of S@Co–N_4_@2D/3D carbon cathode in Li–S battery, the long-term cycle life at a high current density of 1 C with an E/S ratio of 15 µL mg^−1^ was evaluated (Fig. 6f). The S@Co–N_4_@2D/3D carbon could achieve a remarkable capacity of 700 mAh g^−1^ over 500 cycles with a low capacity fading of 0.053% per cycles, which is not only better than S@Co–N_4_@carbon and S@2D carbon cathodes but also comparable to the state-of-the-art reported metal-based compounds/C/S cathodes (Table S2).

To further reveal the superiority of Co–N_4_@2D/3D for suppressing the shuttle effect, lithium foil anodes extracted from cells after 100 cycles at 1 C were investigated. As shown in Fig. S15, the surface of lithium foil in Co–N_4_@2D/3D cells was flat and very smooth, indicating a minimal accumulation of lithium dendrites. However, the surface of Li anode in S@2D carbon- and S@Co–N_4_@carbon cells have been severely corroded. The above results further demonstrate that Co–N_4_@2D/3D has the stronger inhibiting effect for shuttle effect of intermediate lithium polysulfides.

The effect of different SiO_2_ contents on the morphology, specific surface area and pore volume of porous 3D carbon materials was also investigated. For convenience, the samples obtained using 0.75, 1.5, and 3.0 mL tetraethyl orthosilicate were named Co–N_4_@2D/3D carbon-0.75, Co–N_4_@2D/3D carbon, and Co–N_4_@2D/3D carbon-3, respectively. As shown in Fig. S16a, b, Co–N_4_@2D/3D carbon-0.75 and Co–N_4_@2D/3D carbon-3 exhibit a similar morphology to Co–N_4_@2D/3D carbon. The N_2_ sorption measurement results in Fig. S16c indicate that Co–N_4_@2D/3D carbon-0.75 has a Brunauer–Emmett–Teller (BET) surface area of 799 m^2^ g^−1^ and pore volume of 0.718 cm^3^ g^−1^, larger than those of Co–N_4_@2D/3D carbon (488 m^2^ g^−1^ and 0.649 cm^3^ g^−1^) and Co–N_4_@2D/3D carbon-3 (534 m^2^ g^−1^ and 0.56 cm^3^ g^−1^). The largest surface area and pore volume of Co–N_4_@2D/3D carbon-0.75 may be attributed to rich micropores, which is also demonstrated by the pore distribution (Fig. S16d). Moreover, Co–N_4_@2D/3D carbon-0.75, Co–N_4_@2D/3D carbon, and Co–N_4_@2D/3D carbon-3 were used as sulfur host and accordingly their electrochemical performance was also evaluated. As shown in Fig. S17, Co–N_4_@2D/3D carbon-0.75- and Co–N_4_@2D/3D carbon-3-based electrodes could maintain capacities of 839 and 756 mAh g^−1^ over 200 cycles at 0.5 C, respectively, lower than that of Co–N_4_@2D/3D carbon-based electrodes.

## Conclusions

In summary, we have developed a facile strategy for the synthesis of a hierarchically porous three-dimension (3D) carbon architecture assembled by cross-linked carbon leaves with highly dispersed atomic Co–N_4_ through a pyrolysis of SiO_2_-mediated ZIF-L. The porous hierarchical architecture not only effectively buffers the volume change during sulfur lithiation but also provides fast ionic/electronic conductive network for efficient charge transfer. More importantly, the highly accessible Co–N_4_ within 2D/3D carbon functions as an efficient polysulfide regulator to strongly anchor the intermediates against shuttling behavior as well as catalysts to accelerate the sulfur conversion reaction by reducing energy barrier. Benefiting from the structural superiorities, the Li–S cell based on the as-synthesized Co–N_4_@2D/3D carbon exhibited a high sulfur utilization (1171 mAh g^−1^ at 0.2 C), excellent long-term cycling stability (a low capacity fading of 0.053% per cycles for 500 cycles at 1C) and exceptional rate capability (695 mAh g^−1^ at 5 C). Our work may pave a new avenue to develop advanced sulfur host materials for practical Li–S batteries.

## Supplementary Information

Below is the link to the electronic supplementary material.

Supplementary file1 (PDF 1677 kb)
