# Supplementary material for: Porous Carbon Architecture Assembled by Cross-Linked Carbon Leaves with Implanted Atomic Cobalt for High-Performance Li–S Batteries
Source: Nanomicro Lett. 2021 Jun 30;13:151. doi: 10.1007/s40820-021-00676-6 (PMC8245650; doi:10.1007/s40820-021-00676-6)
Supplement: Supplementary file 1 — Supplementary file1 (PDF 1677 kb) [file 40820_2021_676_MOESM1_ESM.pdf]

Supporting Information for

Porous Carbon Architecture Assembled by Cross-Linked Carbon Leaves with Implanted Atomic Cobalt for High-Performance Li-S Batteries

Ruirui Wang[1], Renbing Wu[1, *], Chaofan Ding[1], Ziliang Chen[1], Hongbin Xu[1], Yongfeng Liu[2, *], Jichao Zhang[3], Yuan Ha[1], Ben Fei[1], Hongge Pan[2, 4, *]

[1]Department of Materials Science, Fudan University, Shanghai 200433, P. R. China

[2]State Key Laboratory of Silicon Materials and School of Materials Science and Engineering, Zhejiang University, Hangzhou 310027, P. R. China

[3]Shanghai Synchrotron Radiation Facility, Zhangjiang Laboratory, Shanghai Advanced Research Institute, Chinese Academy of Sciences, Shanghai 201204, P. R. China

[4]Institute of Science and Technology for New Energy, Xi'an Technological University, Xi'an 710021, P. R. China

*Corresponding authors. E-mail: rbwu@fudan.edu.cn (R. Wu), mselyf@zju.edu.cn (Y. Liu); honggepan@zju.edu.cn (H. Pan)

Supplementary Figures and Tables

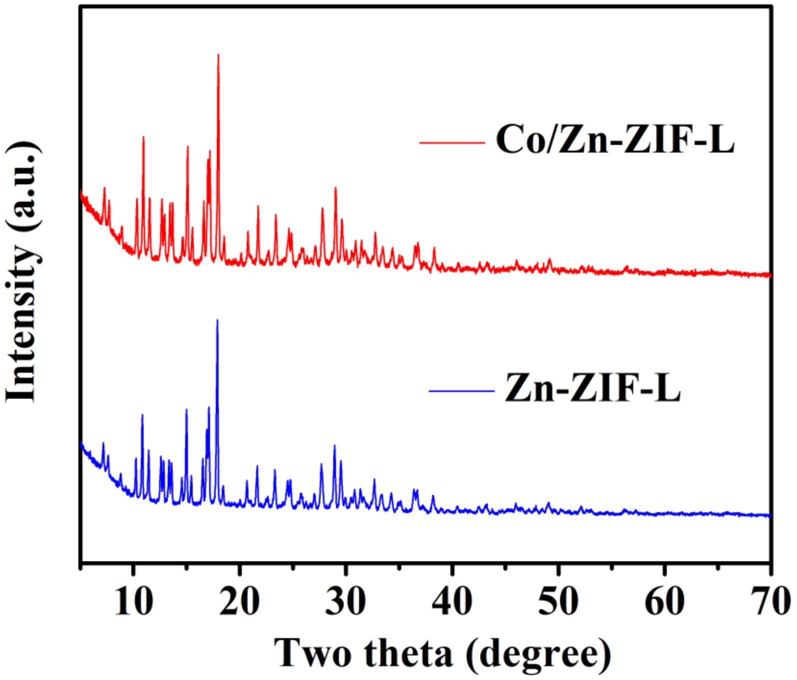

Fig. S1 XRD patterns of Co/Zn-ZIF-L and Zn-ZIF-L

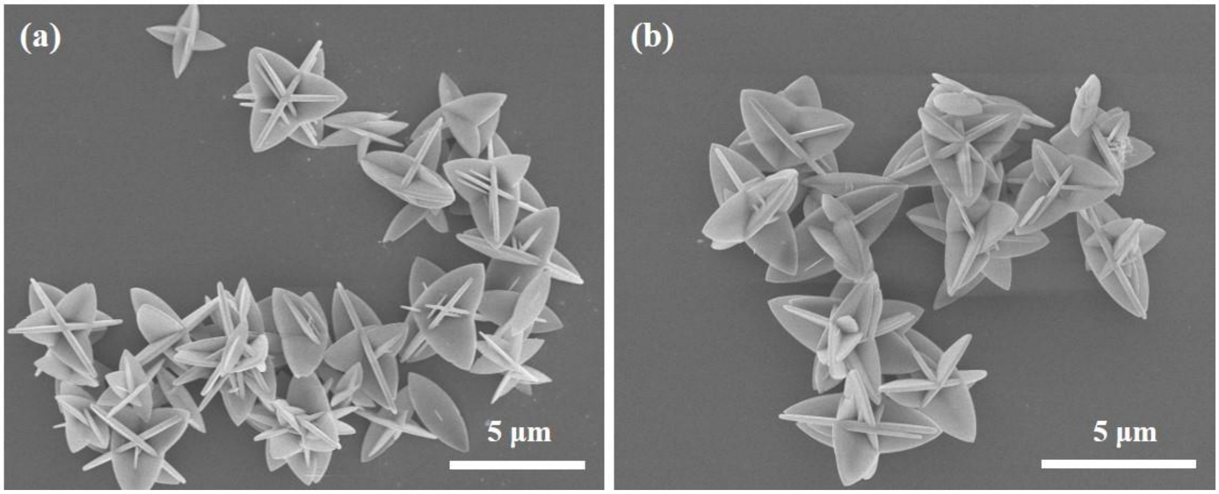

Fig. S2 a, b FESEM images of Co/Zn-ZIF-L

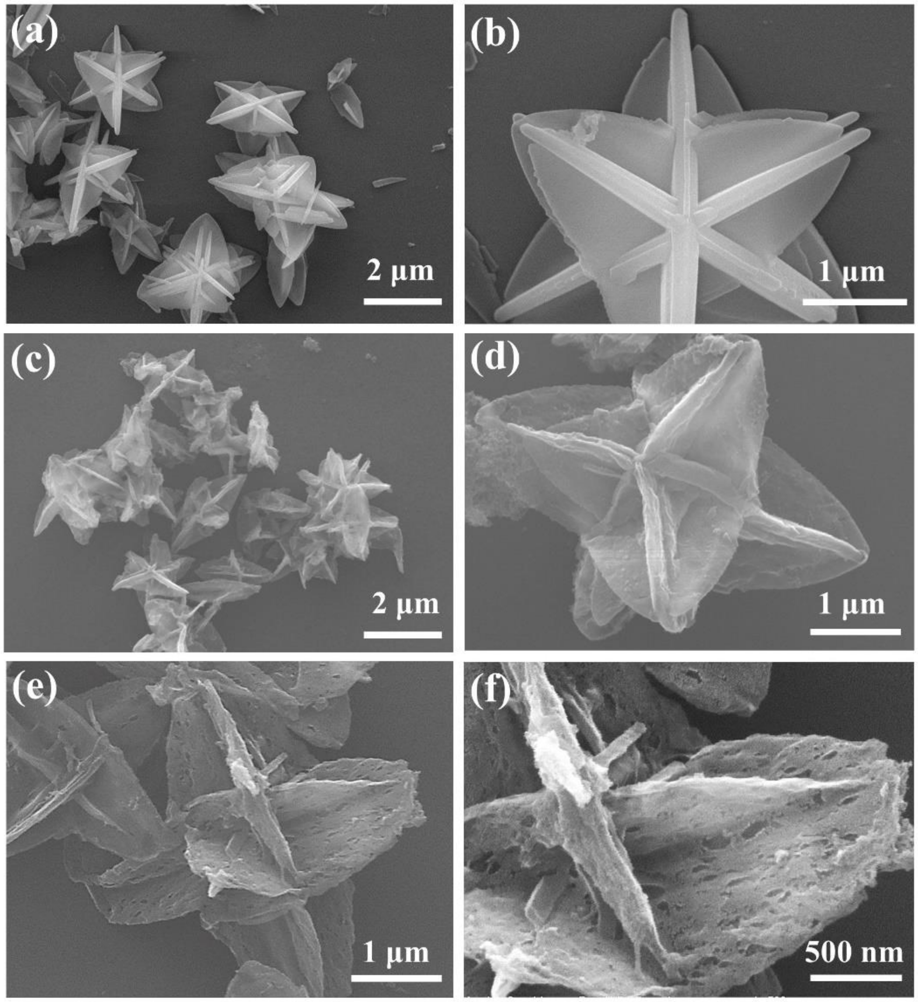

Fig. S3 a, b FESEM images of Co/Zn-ZIF-L; **c, d** FESEM images of Co-N_4@2D/3D carbon@SiO_2; **e** and **f** FESEM images of Co-N_4@2D/3D carbon

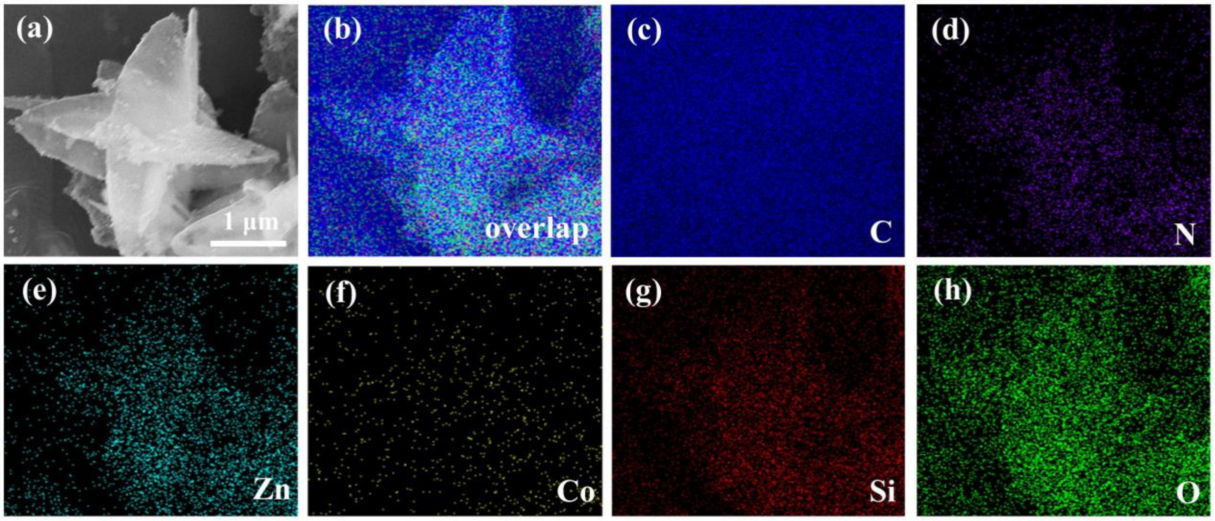

Fig. S4 a FESEM image of Co/Zn-ZIF-L@SiO_2; **b-h** corresponding EDS elemental mapping of Co/Zn-ZIF-L@SiO_2

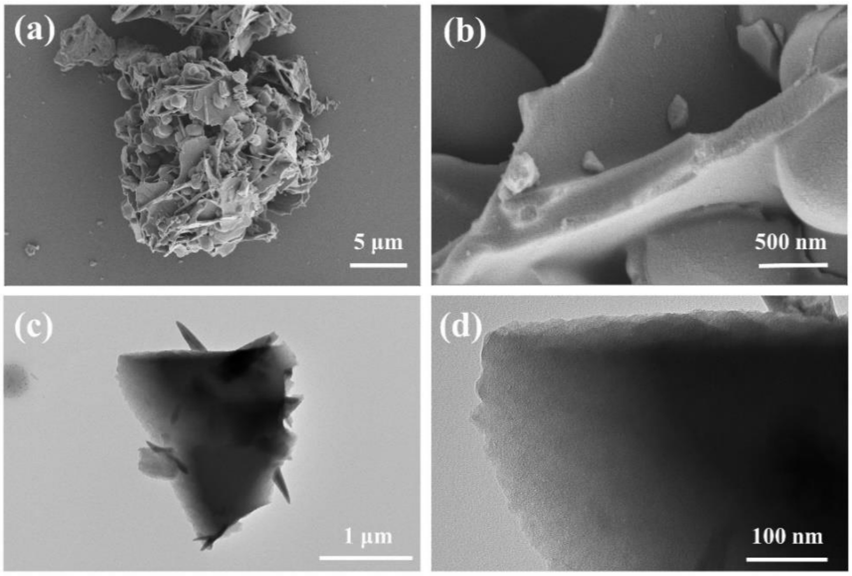

Fig. S5 a, b FESEM and **c, d** TEM images of Co-N_4@carbon

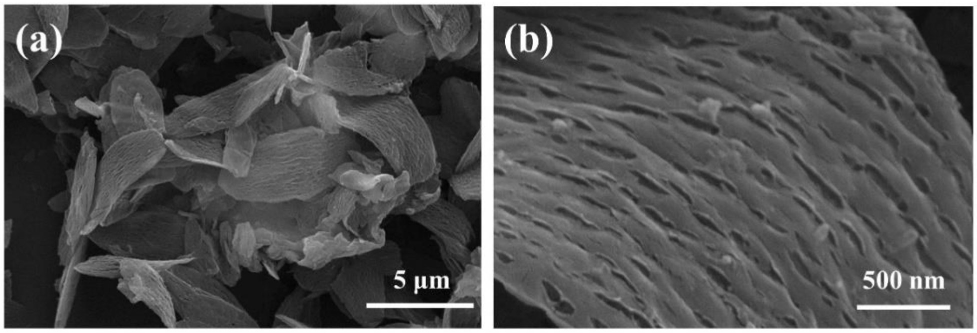

Fig. S6 a Low- and **b** high-magnification FESEM images of 2D carbon.

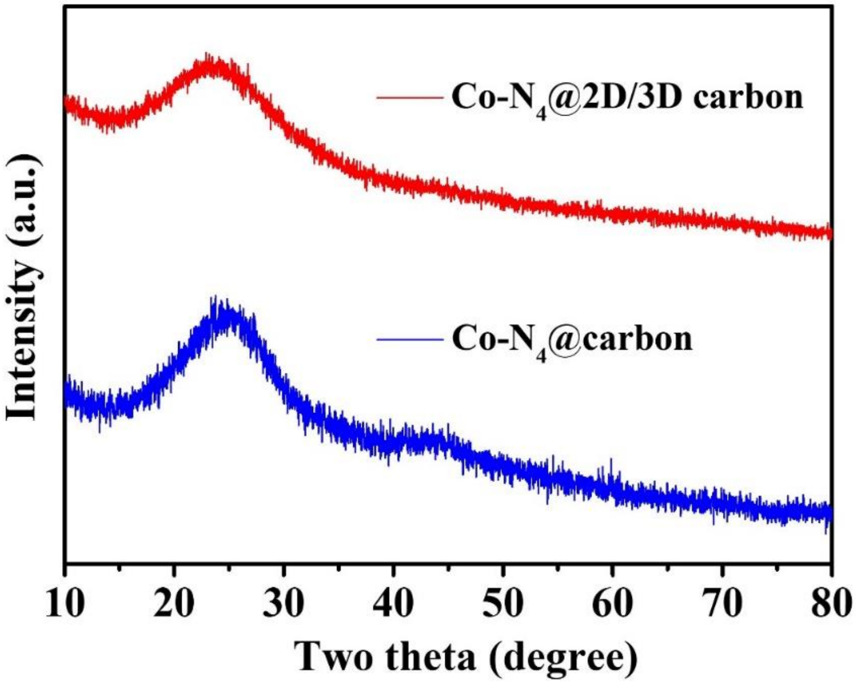

Fig. S7 XRD patterns of Co-N_4@2D/3D carbon and Co-N_4@carbon

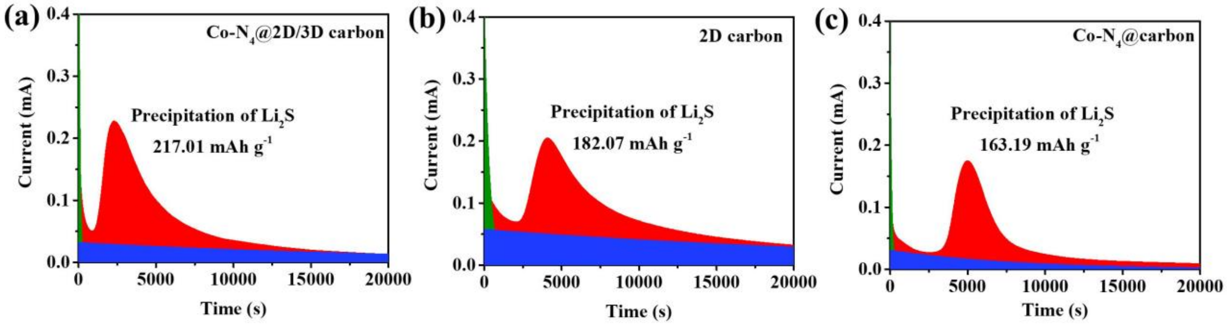

Fig. S8 Potentiostatic discharge profiles at 2.05 V with Li_2S_8 catholyte of **a** Co-N_4@2D/3D carbon, **b** S@2D carbon and **c** Co-N_4@carbon

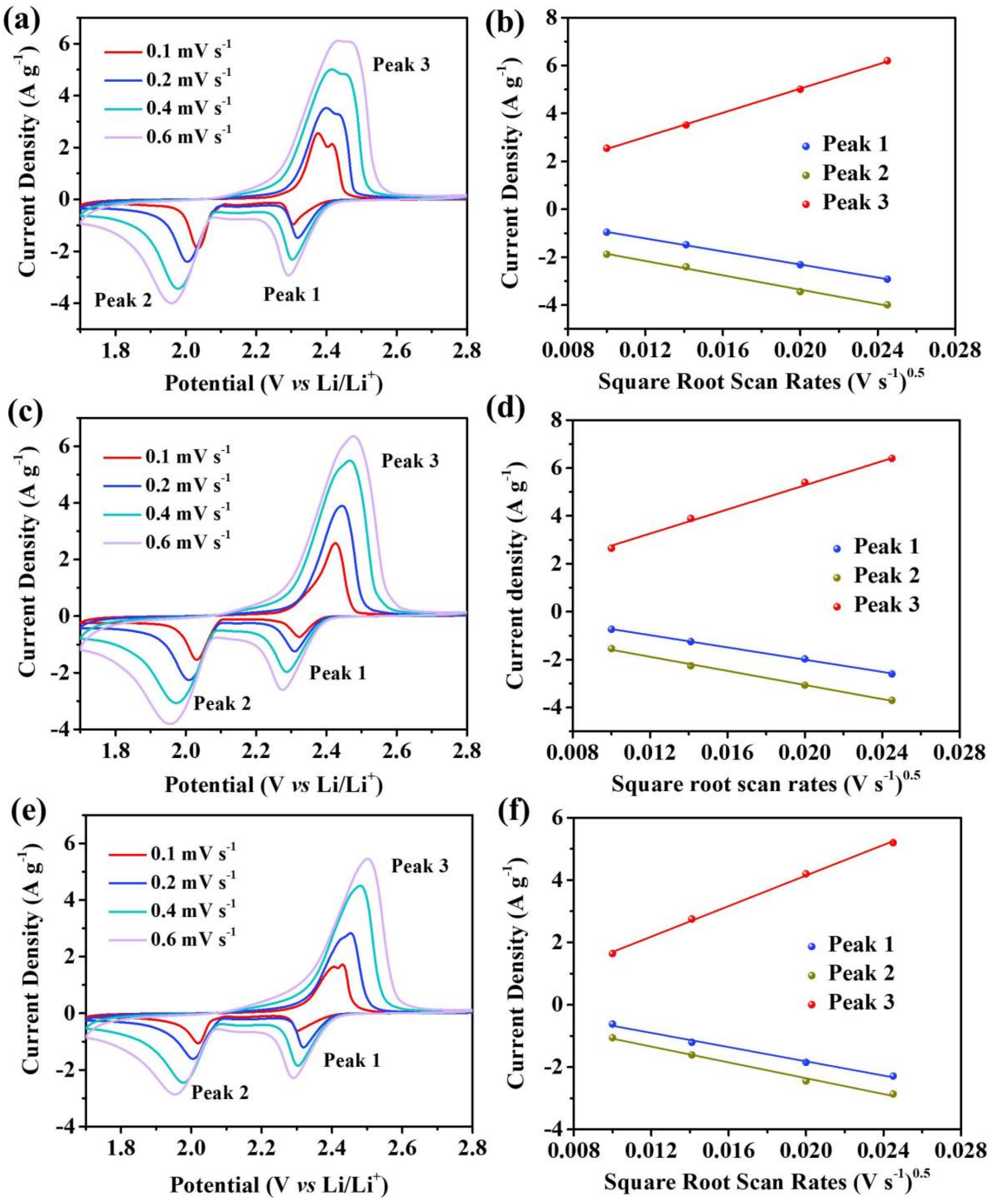

Fig. S9 a, c and **e** CV curves of S@Co-N_4@2D/3D carbon, S@2D carbon and S@Co-N_4@carbon cathodes at different scan rates in the range of 0.1−0.6 mV s^{-1}, respectively. **b, d** and **f** The fitting lines between peak currents and the square root of the scan rates

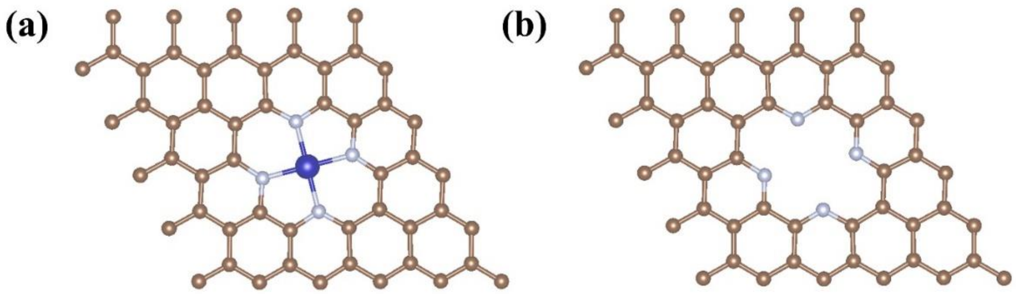

Fig. S10 Optimized structures of **a** Co-N4@2D/3D carbon and **b** 2D carbon

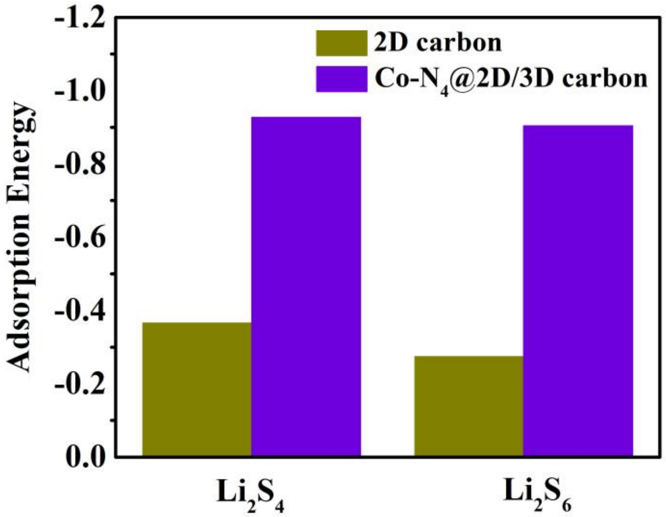

Fig. S11 Adsorption energy of Co-N4@2D/3D carbon and 2D carbon for Li_2S_4 and Li_2S_6, respectively

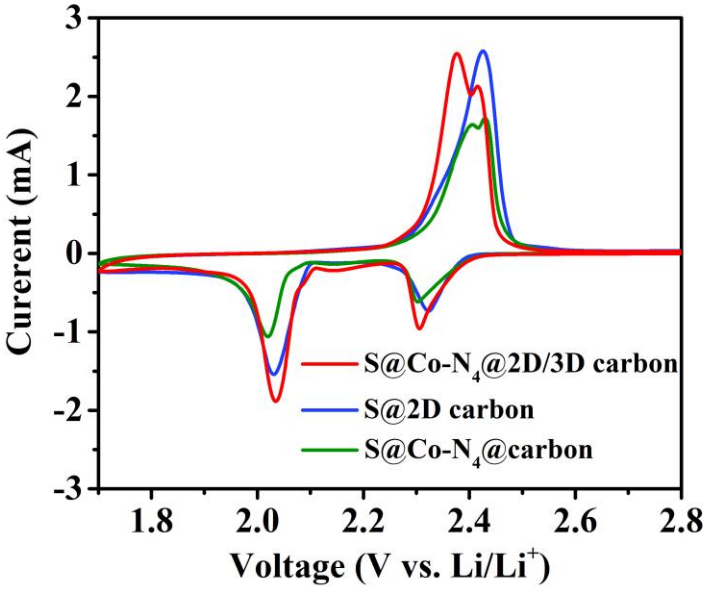

Fig. S12 CV curves of S@Co-N4@2D/3D carbon, S@Co-N4@carbon and S@2D carbon cathodes within a potential window of 1.7−2.8 V at a scan rate of 0.1 mV s^{-1}

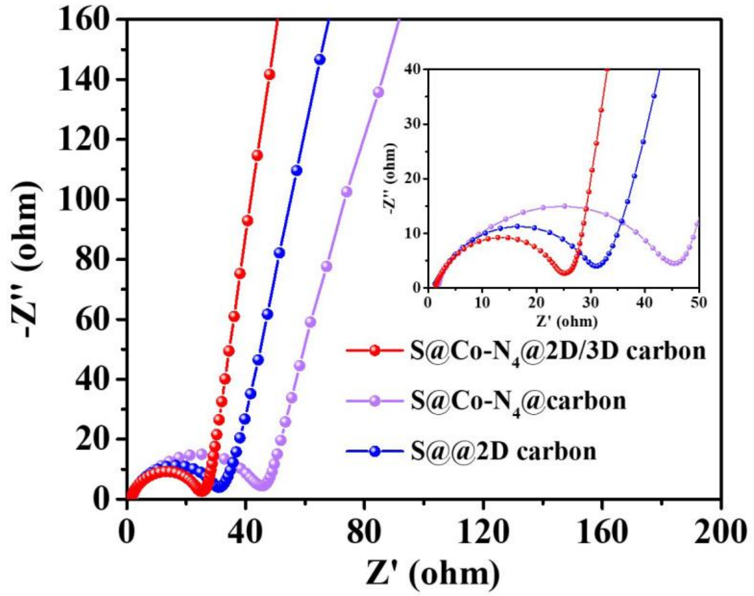

Fig. S13 EIS spectra of S@Co-N$_4$@2D/3D carbon, S@Co-N$_4$@carbon and S@2D carbon electrodes

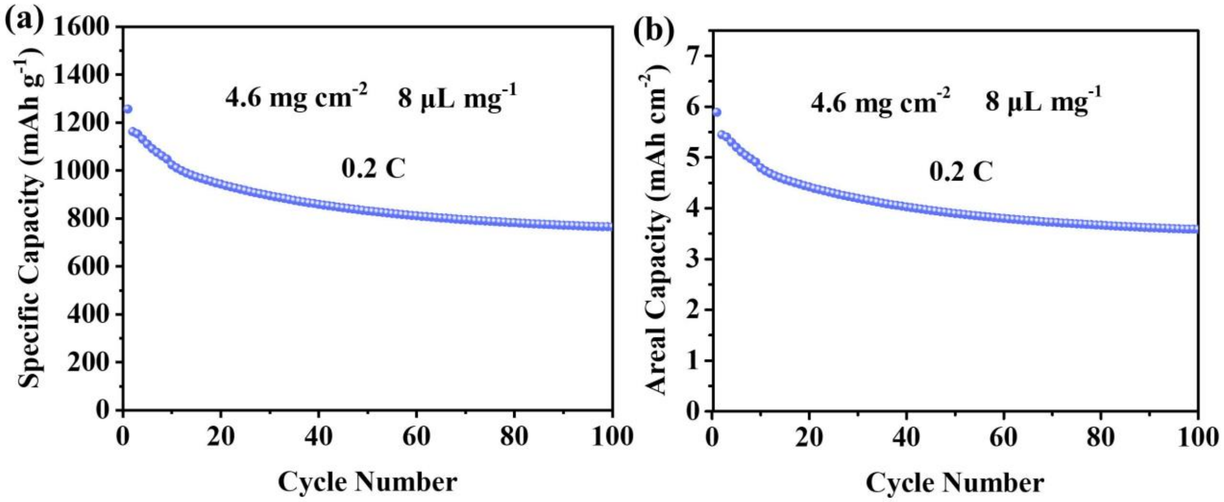

Fig. S14 Cycling performances of S@Co-N$_4$@2D/3D carbon cathode at 0.2 C with high-loading sulfur and low E/S ratio

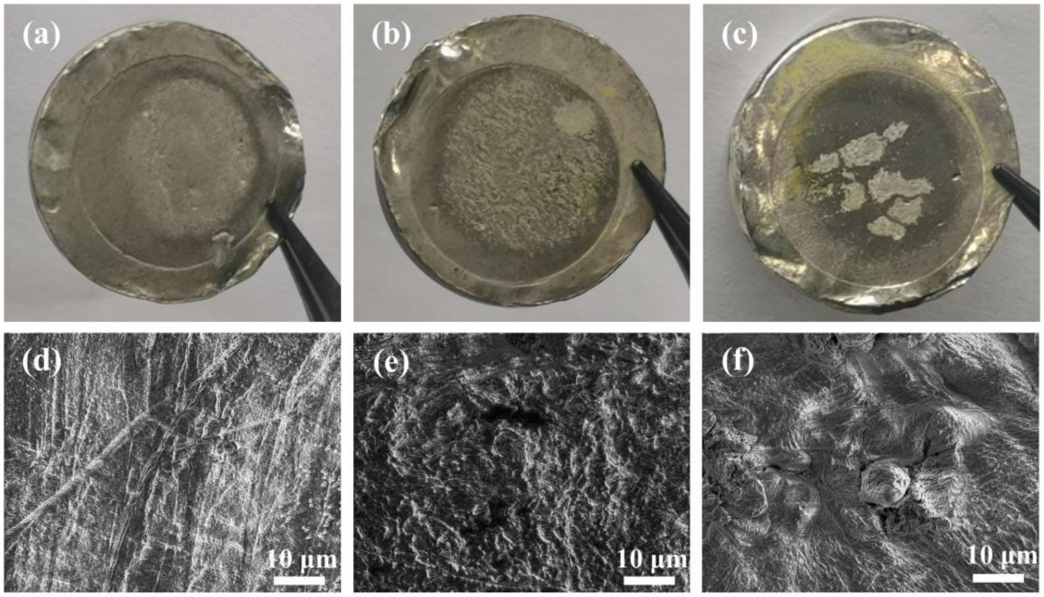

Fig. S15 Optical and SEM images of the recovered lithium foils paired with **a and d** S@Co-N_4@2D/3D carbon cathode, **b and e** S@2Dcarbon cathode, **c and f** S@Co-N_4@carbon cathode after 100 cycles at 1 C

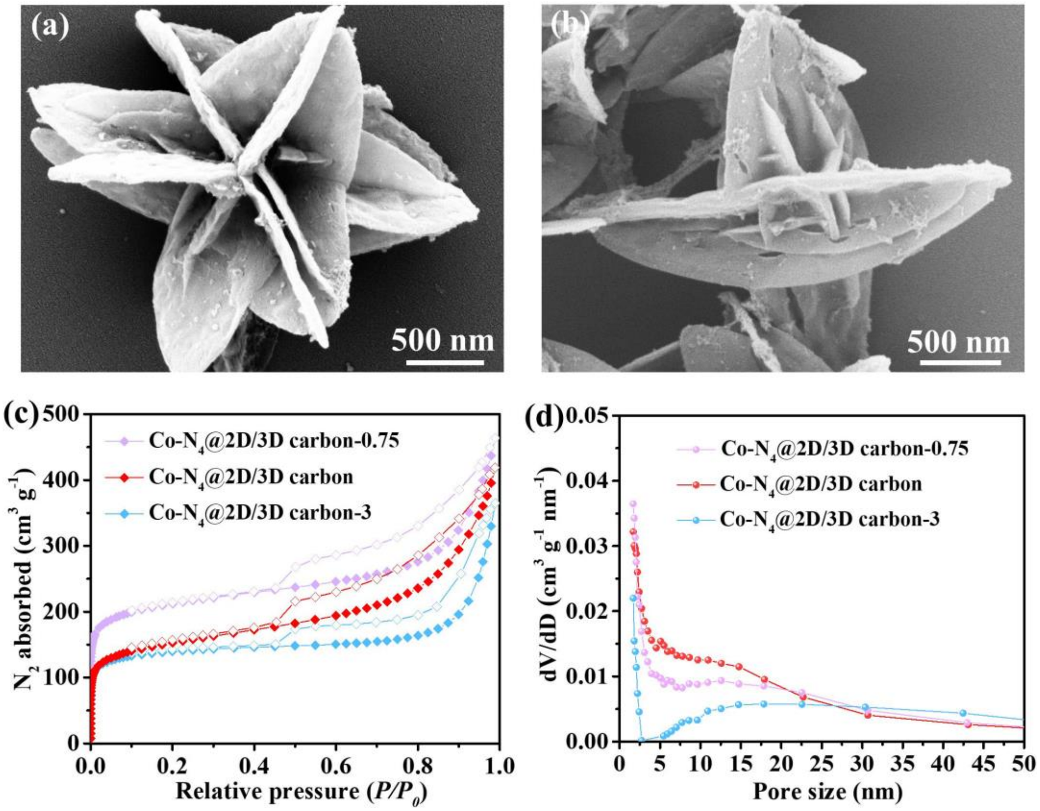

Fig. S16 FESEM images of **a** Co-N_4@2D/3D carbon-0.75 and **b** Co-N_4@2D/3D carbon-3. **c** N_2 sorption isotherms based on the Barrett-Joyner-Halenda (BJH) method and **d** the pores distribution of Co-N_4@2D/3D carbon-0.75, Co-N_4@2D/3D carbon and Co-N_4@2D/3D carbon-3

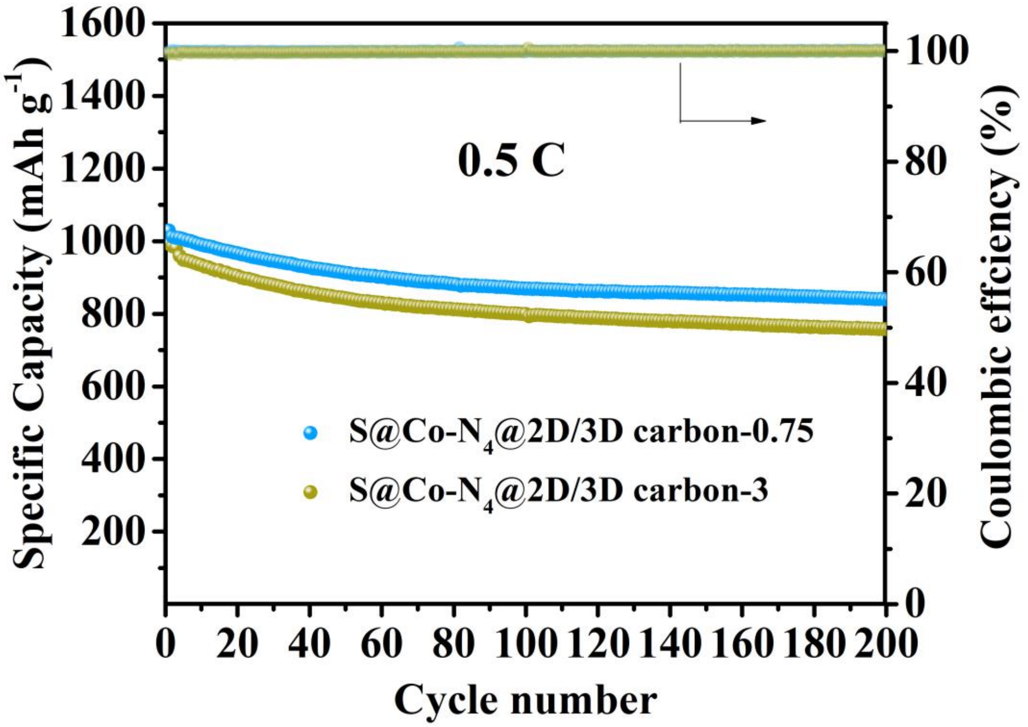

Fig. S17 Cycling performance of cathode based on Co-N_4@2D/3D-0.75 and Co-N_4@2D/3D-3 at 0.5 C

Table S1 Co K Edge XAS fitting parameters of Co-N_4@2D/3D carbon

Material	Shell	N	R(Å)	σ^2(Å^2)	ΔE_0(eV)
Co-N_4@2D/3D carbon	Co-N	4.081	1.90002	0.00995	-4.363

Table S2 Comparison of electrochemical performances of S@Co-N_4@2D/3D carbon composites electrode with previously reported metal-based compounds/C/S electrodes

Composites	Sulfur content	Rate performance (A/B)	Cycling stability(A/B/n)	Refs.
S@Co-N_4@2D/3D carbon	73%	805/2C<br>695/5C	0.053/1/500	This work
S@MoC@N-CNF	50%	799/2C	0.084/1/350	[S1]
S@Co/N-PCNSs	68%	683/2C	0.10/1/500	[S2]
S/N-CNTs/Co-NFs	78%	684/3C	0.078/1/500	[S3]

S@H-Co-NCM	82%	611/2C	0.069/0.5/500	[S4]
CoS_2@ NGCNs/S	75%	525.5/2C	0.078/1/300	[S5]
NC/MoS_3-S	70%	596.7/3C	0.076/0.5/500	[S6]
S@Co-NBC	77.2%	509/5C	0.09/1/500	[S7]
S@Co@BNCNTs YS microspheres	64%	752/2C	0.06/1/400	[S8]
S/Co-NPC-MCs	80%	752/2C	0.07/1/400	[S9]
CC@Co-CNAs/Li_2S_6	66%	807/1C	409/1/500	[S10]

A/B means the capacity of A (mAh g^{-1}) at the certain rate current density of B.

A/B/n means the capacity of A (mAh g^{-1}) at the certain rate current density of B after cycles of n.

Supplementary References

[S1] H.F. Shi, Z.H. Sun, W. Lv, S.G. Wang, Y. Shi et al., Necklace-like MoC sulfiphilic sites embedded in interconnected carbon networks for Li-S batteries with high sulfur loading. J. Mater. Chem. A **7**, 11298–11304 (2019). https://doi.org/10.1039/C9TA00741E

[S2] S.H. Liu, J. Li, X. Yan, Q.F. Su, Y.H. Lu et al., Superhierarchical Cobalt-embedded nitrogen-doped porous carbon nanosheets as two-in-one hosts for high-performance lithium-sulfur batteries. Adv. Mater. **30**, 1706895 (2018). https://doi.org/10.1002/adma.201706895

[S3] L.B. Ma, H.N. Lin, W.J. Zhang, P.Y. Zhao, G.Y. Zhu et al., Nitrogen-doped carbon nanotube forests planted on cobalt nanoflowers as polysulfide mediator for ultralow self-discharge and high areal-capacity lithium-sulfur batteries. Nano Lett. **18**, 7949–7954 (2018). https://doi.org/10.1021/acs.nanolett.8b03906

[S4] S.X. Chen, X.X. Han, J.H. Luo, J. Liao, J. Wang et al., In situ transformation of LDH into hollow cobalt-embedded and N-doped carbonaceous microflowers as polysulfide mediator for lithium-sulfur batteries. Chem. Eng. J. **385**, 123457 (2020). https://doi.org/10.1016/j.cej.2019.123457

[S5] S.D. Seo, D. Park, S. Park, D.W. Kim, "Brain-coral-like" mesoporous hollow CoS_2@N-doped graphitic carbon nanoshells as effcient sulfur reservoirs for lithium-sulfur batteries. Adv. Funct. Mater. **29**, (2019) 1903712. https://doi.org/10.1002/adfm.201903712

[S6] J. Yu, J.W. Xiao, A.R. Li, Z. Yang, L. Zeng et al., Enhanced multiple anchoring and catalytic conversion of polysulfides by amorphous MoS_3 nanoboxes for high-

performance Li-S batteries. Angew. Chem. Int. Ed. **59** 13071–13078 (2020). https://doi.org/10.1002/anie.202004914

[S7] Z. Zhang, D.G. Xiong, A.H. Shao, X.Y. Huang, Y. Huang et al., Integrating metallic cobalt and N/B heteroatoms into porous carbon nanosheets as efficient sulfur immobilizer for lithium-sulfur batteries. Carbon **167**, 918–929 (2020). https://doi.org/10.1016/j.carbon.2020.06.017

[S8] S.K. Park, J.K. Lee, Y.C. Kang, Yolk-shell structured assembly of bamboo-like nitrogen-doped carbon nanotubes embedded with Co nanocrystals and their application as cathode material for Li-S batteries. Adv. Funct. Mater. **28**, 1705264 (2018). https://doi.org/10.1002/adfm.201705264

[S9] P. Wang, Z. Zhang, X.L. Yan, M. Xu, Y.X. Chen et al., Pomegranate-like microclusters organized by ultrafine Co nanoparticles@nitrogen-doped carbon subunits as sulfur hosts for long-life lithium-sulfur batteries. J. Mater. Chem. A **6**, 14178–14187 (2018). https://doi.org/10.1039/C8TA04214D

[S10] H. Zhang, L.G. Wang, Q. Li, L. Ma, T.P. Wu et al., Cobalt nanoparticle-encapsulated carbon nanowire arrays: enabling the fast redox reaction kinetics of lithium-sulfur batteries. Carbon **140**, 385–393 (2018). https://doi.org/10.1016/j.carbon.2018.09.012
